# The Shortage of Resident Medical Officers

**Published:** 1915-08-07

**Authors:** 


### THE BRITISH HOSPITALS ASSOCIATION.

### The Shortage of Resident Medical Officers.

The following is the substance of the paper read by Mr. J. C. Buchanan, Secretary, Metropolitan Hospital:—

The difficulty in obtaining the full complement of resident medical officers is believed to be common to nearly all the hospitals. Serious as it was before the war, it has increased owing to and during the war, and it is likely to attain more serious proportions still. Inquiries appertaining to the subject under discussion have been addressed to fifty hospitals. The answers which have been returned reveal the fact that the expenditure on residents is very greatly in excess of that which is incurred in normal times, but that no hospital is at the present moment in extreme difficulty. The ordinary economic law of supply and demand applies, and as the demand for resident medical officers is considerably in excess of the supply, hospitals have to pay more for their professional services. Hospitals which require only two or three residents have been able up to now to fill the vacancies in the junior staff as they have arisen, though, as just indicated, the salaries payable have been very largely increased.

Those hospitals which require four, five, and six residents (not teaching hospitals) have experienced just that proportionately greater difficulty in filling their junior appointments. Some alteration in the work of the hospitals has in many cases been necessary, but in no case so far has the work been to any serious extent curtailed. Generally speaking, the in-patient accommodation that is normally available remains available. Such alterations as have been made have been of some such nature as the following. In special out-patient departments which have been previously open on several days a week the work has been limited to one or two days a week. Out-patients have been seen only in cases of urgency or on the recommendation of a general practitioner. The rules applying to insurance cases have been more strictly enforced. In many hospitals lady doctors have been appointed. In some cases this is a complete innovation. A further alteration in the work of many hospitals—and in their number is to be included the Metropolitan—is as regards the casualty work. This has been carried out by medical men practising in the neighbourhood prevented from joining the Royal Army Medical Corps. This arrangement has worked with complete success and pleasantly for all concerned.

The difficulty at the hospitals without medical schools has already been very serious indeed. It would be a shame to lose any opportunity of acknowledging that this difficulty would have been very much more serious but for the loyalty of the visiting staff. Senior members of the visiting staff of long-standing consulting practice, regardless of their personal interest, have taken their turns as they came round to sleep at the hospital, once more resuming the house physicianship or house surgency they relinquished many years ago.

For the hospitals with medical schools attached a scheme has been arranged by the War Office for granting early commissions to resident medical officers. All those connected with the teaching hospitals are thoroughly familiar with the scheme; and as, according to present information, it is to be applied rigidly and only to teaching hospitals, it is not necessary to deal with it at any length. [*Cf.* The Hospital, July 17, p. 323.]

The difficulty, as far as the teaching hospitals are concerned, is practically met. In hospitals where before this scheme there were not sufficient applications for the appointments vacant, it is anticipated that there will be a fairly large surplus of applications.

It is the position in hospitals without medical schools, to which this scheme does not apply, which is most in jeopardy. For it cannot be expected that any self-respecting young man will without some special reason desire to go on working at a hospital in obscurity at a salary of between £150 to £200 a year when he sees his professional brethren in khaki uniform in receipt of a

### The British Hospitals Association (*continued*).

larger salary. This, in fact, is the position which is before us. Residents are absolutely essential to the efficient discharge of the work in certain departments of a voluntary hospital. Some provision must be definitely made for them.

The number of doctors in existence, however, is limited, and it takes a very much longer time to turn a layman into a doctor than it takes to turn a civilian into a soldier. But this process has also to be gone through by doctors accepting these temporary commissions. The possession of a commission entails its responsibilities like the possession of any other honour. It will be seen that these responsibilities are real.

The question is what sort of attraction to residents or prospective residents a hospital can hold out which will be anything like so effective as the honour and glory of military service, which, be it recollected, is not without considerable material advantages. There are bound to be good reasons for limiting the granting of temporary commissions to residents of teaching hospitals. At the same time it does appear at first sight as if the one and best way out of the difficulty has been closed. It seems a pity to lose any additional training which would in the long run be of such advantage not only to the individual doctors both at and after the war, but also, if the statement may be ventured without impertinence, to the State Medical Service in the interests of the soldiers themselves. While the application of this temporary commission scheme to all hospitals treating, say, a stated number of soldiers would at once secure residents to these hospitals, it would have an additional advantage in allaying fears of disorganisation of which some of the civil hospitals grow conscious.

It has already been stated that the remuneration payable to residents has been increased. The hospitals are in no position to compete with the War Office in the matter of salaries. The attraction for medical officers must be found on some other than material grounds. To take the place of the consulting staff who leave to take up military duties, the retired members of the staff can sometimes be called back into practice. But the work of the resident staff is our pressing difficulty. It might be possible to induce general practitioners in the neighbourhood of hospitals to extend their generosity still further. But general practitioners cannot be expected to leave their homes and practices to spend nights in the hospital, as the visiting staff are doing in some hospitals. Another suggestion is the engagement of graduates of foreign universities, subject to registration by the General Medical Council. This expedient has not up to now been very largely resorted to. Its effective application must depend on an adequate knowledge of the English language, otherwise it would mean confusion worse confounded. Again graduates of Canadian and Australian universities (in many cases senior men with very high English qualifications) have served with great success. Then, again, doctors of the United States might be called upon. The difficulty here is that of medical registration; as in America, there is a great variety of qualifications. There is no reciprocity in this matter between one State and another, nor between England and America.

Civil hospitals have given a great deal of their in-patient accommodation for sick and wounded soldiers. In addition to the accommodation within their own walls, hospitals have undertaken the administration of large public buildings, schools, etc., converted into hospitals and equipped at the expense of the War Office. There is no question that the hospitals are "doing their bit." This fact is generously and graciously acknowledged by the War Office authorities. So much so that it might seem that the hospitals have almost established some sort of right, in doing the work for soldiers, to special consideration in the matter of residents; and there is little doubt but that some solution of the present difficulty will be found.

